# Analyzing mRNAsi-Related Genes Identifies Novel Prognostic Markers and Potential Drug Combination for Patients with Basal Breast Cancer

**DOI:** 10.1155/2021/4731349

**Published:** 2021-10-04

**Authors:** Kai Huang, Yu Wu, YunQing Xie, LiYing Huang, Hong Liu

**Affiliations:** ^1^The Second Department of Breast Cancer, Tianjin Medical University Cancer Institute and Hospital, National Clinical Research Center for Cancer, Tianjin 300600, China; ^2^Key Laboratory of Cancer Prevention and Therapy, Tianjin 300600, China; ^3^Tianjin's Clinical Research Center for Cancer, Tianjin 300600, China; ^4^Key Laboratory of Breast Cancer Prevention and Therapy, Tianjin Medical University, Ministry of Education, Tianjin 300060, China; ^5^Department of Breast Surgery, Fujian Medical University Cancer Hospital, Fujian Cancer Hospital, Fuzhou, Fujian 350014, China; ^6^Department of Head and Neck Surgery, Fujian Medical University Cancer Hospital, Fujian Cancer Hospital, Fuzhou, Fujian 350014, China; ^7^Department of Surgical Laboratory, Fujian Medical University Cancer Hospital, Fujian Cancer Hospital, Fuzhou, Fujian 350014, China

## Abstract

Basal breast cancer subtype is the worst prognosis subtypes among all breast cancer subtypes. Recently, a new tumor stemness index-mRNAsi is found to be able to measure the degree of oncogenic differentiation of tissues. The mRNAsi involved in a variety of cancer processes is derived from the innovative application of one-class logistic regression (OCLR) machine learning algorithm to the whole genome expression of various stem cells and tumor cells. However, it is largely unknown about mRNAsi in basal breast cancer. Here, we find that basal breast cancer carries the highest mRNAsi among all four subtypes of breast cancer, especially 385 mRNAsi-related genes are positively related to the high mRNAsi value in basal breast cancer. This high mRNAsi is also closely related to active cell cycle, DNA replication, and metabolic reprogramming in basal breast cancer. Intriguingly, in the 385 genes, *TRIM59*, *SEPT3*, *RAD51AP1*, and *EXO1* can act as independent protective prognostic factors, but *CTSF* and *ABHD4B* can serve as independent bad prognostic factors in patients with basal breast cancer. Remarkably, we establish a robust prognostic model containing the 6 mRNAsi-related genes that can effectively predict the survival rate of patients with the basal breast cancer subtype. Finally, the drug sensitivity analysis reveals that some drug combinations may be effectively against basal breast cancer via targeting the mRNAsi-related genes. Taken together, our study not only identifies novel prognostic biomarkers for basal breast cancers but also provides the drug sensitivity data by establishing an mRNAsi-related prognostic model.

## 1. Introduction

Breast cancer is the most serious primary malignant tumor type in women, and its incidence rate is about 1.3 million, and the death rate is about 0.5 million worldwide [1, 2]. Some certain factors, such as mental pressure, large amount of estrogen secretion, and late childbearing, have led to the onset of breast cancer in younger women with the sustained economic and social development [1, 2]. Currently, the main treatment measures of breast cancer are surgery combined with radiotherapy, chemotherapy, endocrine therapy, and targeted drug therapy, but the curative effect and prognosis for patients are not satisfactory [3, 4]. Breast cancer is a highly heterogeneous tumor. Especially, according to immunohistochemical markers, breast cancer patients can be divided into four subtypes: luminal A, luminal B, HER2, and basal. Among them, the basal subtype is commonly referred to as triple-negative breast cancer (PR-, ER-, HER2-), which usually has the worst prognosis [3, 5]. In fact, the basal subtype also contains multiple different molecular patterns. A study demonstrated that the basal subtype could be composed of at least two clinically distinct groups, i.e., poor or better survival rate [6]. Another study revealed the basal subtype could be divided into two types (basal-like 1, basal-like 2). The basal 1 contains abundant cell cycle and cell division components, but the basal 2 includes growth factor signaling, glycolysis, and gluconeogenesis pathways [7]. Alternatively, the basal subtype was further defined the Basal-Like Immune-Suppressed (BLIS) and the Basal-Like Immune-Activated (BLIA) subtype [8]. All these above studies indicated that the basal subtype is a very heterogeneous breast tumor. Therefore, clearly distinguishing molecular subtype is crucially essential to elucidate the biological and clinical characteristics of the basal subtype as well as to establish the personalized treatment measures for breast cancer patients.

The tumor microenvironment is complex and diverse, which is composed of relatively differentiated cancer cells, tumor stem cells, endothelial cells, tumor-related fibroblasts, invasive immune cells, and other cell types or components [9]. Notably, not all cells play a role in the occurrence, metastasis, and recurrence of tumor in the tumor microenvironment. Only a few tumor cells could be endowed with stem cell characteristics and invasive ability, and these cells not only have strong tumorigenicity but can escape the recognition of the body's immune system, thereby inducing innate resistance to external killing. These cells are called cancer stem cells (CSCs) which can be induced by some certain environmental factors. The CSCs are an important part of the tumor microenvironment, which not only have the characteristics of continuous proliferation of cancer cells but have the characteristics of self-renewal and multidirectional differentiation [10, 11].

Many studies have revealed that breast cancer stem cells can produce a large number of primary tumors, thereby driving tumor occurrence and metastasis to lead to the poor prognosis for breast cancer patients [12, 13]. Especially, a recent study has determined that the undifferentiated cell population with stem cell like characteristics is the main factor affecting the recurrence and progression of breast cancer [14]. Remarkably, several breast cancer CSC markers, such as CD44, CD24, ALDH1, CD133, CD29, CD61, DLL1, PROCR, MUC1, THY1, and GATA3, have been identified [12, 15]. In particular, CD44, CD24, and ALDH1 have been considered to have potential prognostic effects in triple-negative breast cancer (TNBC) subtypes [16, 17] and may act as certain clinical metrics, such as recurrence, distant metastasis, disease-free survival, and overall survival [18]. Fascinatingly, a new method of describing tumor stem cells called as tumor stemness features has been established by using artificial intelligence and deep learning algorithms [19, 20]. Tumor stemness features can be used to quantify the gradual loss of differentiated phenotypes and the acquisition of progenitor cells and stem cell-like features during the progression of cancer [19, 20]. For example, Malta et al. have innovatively applied one class logistic regression (OCLR) machine learning algorithm to various types of stem cells and tumor cells to extract the expression profile characteristics between them [19]. Especially, they have used the algorithm and features to the genome-wide expression data of tens of thousands of TCGA samples for quantifying the degree of tissue carcinogenic differentiation [19]. This work eventually led them to propose a new tumor stemness index mRNAsi for describing the degree of similarity between tumor cells and stem cells [19]. The mRNAsi value range is from 0 to 1, and the mRNAsi score is significantly correlated with tumor dedifferentiation level and biological process of tumor stem cells [19]. Studies have suggested that mRNAsi might serve as an effective index for the survival, classification, and disease progression of tumor patients [21–24]. Taken together, these studies provide a new insight for further revealing the mechanism of breast cancer stem cell of the basal-like subtype.

In this study, we firstly determined the mRNAsi of each breast cancer subtype and found that the mRNAsi value of the basal subtype was the highest among all subtypes. We thereby determined to investigate the role of mRNAsi in the patients with the basal subtype. We used the WGCNA to screen key genes related to mRNAsi in the patients with the basal subtype. We next explored the potential functions of these mRNAsi-related genes in the basal breast cancer patients. Our results demonstrated that cell cycle might have an important role in the occurrence and development of breast cancer patients of the basal subtype. Especially, we developed a prognostic model based on mRNAsi for breast cancer patients of the basal subtype. The robust prognostic model consists of 6 genes and has a good predictive performance. In conclusion, our work revealed that mRNAsi is highly correlated with the basal subtype; in particular, these mRNAsi-related genes can be hoped to serve as biomarkers for clinical prognosis and therapy of breast cancer patients of the basal subtype.

## 2. Materials and Method

### 2.1. The Acquisition and Processing of Data

The mRNAsi of breast cancer based on pluripotent stem cell samples was obtained from the work of Malta et al. [19]. Gene expression profiles of the basal subtype samples and corresponding clinical information were originated from UCSC Xena database (http://xena.ucsc.edu/). The selected samples were adopted by following criteria: (1) clinical data such as overall survival days and survival status are complete; (2) normal samples were selected only for the matching pairs of cancer tissue. After filtering, 185 breast cancer samples and 17 normal tissue samples with both normalized expression fragments per kilobase million (FPKM) and clinical data were retained for next analysis. Differentially expressed genes (DEGs) were screened using the limma R package [25]; the filtering criteria were ∣log2FC | >1 and FDR < 0.05. The drug sensitivity data of the candidate prognostic marker came from GSCALite database (http://bioinfo.life.hust.edu.cn/web/GSCALite/). The modified database has integrated the genome-wide drug sensitivity test data of CTRP and GDSC and provides a good resource for exploring the sensitivity of genes to drugs.

### 2.2. The Establishment of WGCNA

The Weighted Gene Coexpression Network Analysis (WGCNA) is a system biology method of describing gene association expression patterns among different samples and has been widely used to identify highly collaborative gene sets and alternative biomarker genes or therapeutic targets according to the connectivity of gene sets and the association between gene sets and phenotypes [26]. Here, we selected differentially expressed genes between breast cancer and normal samples for WGCNA R package for gene module analysis [27]. The specific analysis process was as follows: (1) removing outlier samples, (2) calculating soft thresholds to fit the best scale-free network, (3) constructing a gene dissimilarity matrix, and (4) identifying coexpression modules and calculating module feature vectors. After we obtained the different gene module, we selected mRNAsi as the clinical phenotypes and then analyzed the module in combination with the clinical phenotype; the absolute value of correlation coefficient greater than 0.3 is considered to be correlated, and a *P* value < 0.05 indicates that the correlation is statistically significant.

### 2.3. Tumor Immune Infiltration Cell Transformation

The single sample gene set enrichment analysis (ssGSEA) strategy was used to evaluate genome-wide expression and tumor immune cell infiltration distribution based on 782 marker genes of 28 tumor immune infiltrating cells (TIICs) [28]. Herein, we used the GSVA package to convert mRNA expression data into the enrichment score of 28 tumor immune infiltrating cells (TIICs) of patients [29].

### 2.4. Somatic Mutation Analysis

The somatic mutation map of the basal breast cancer patients based on the whole exome sequencing platform was downloaded from GDC (https://portal.gdc.cancer.gov/), and the R package maftools was used to summarize and analyze the data [30]. Genome mutation types include missense mutation, frame shift deletion mutation, nonsense mutation, frame shift insertion mutation, splice site, in-frame insertion and in-frame samples with deletion, transcription start site, and stop codon mutation that were considered mutation positive.

### 2.5. Function Analysis of Methylation-Driven Genes

Both Gene Ontology (GO) and Kyoto Encyclopedia of Genes and Genomes (KEGG) analyses were carried out to reveal these functional roles of genes associated with mRNAsi by clusterProfiler R package [31], and *P* < 0.05 is considered significant unless otherwise noted.

### 2.6. The Establishment of Prognosis Model

MRNAsi-related genes were used to construct a prognostic model, and the construction method was as follows: the univariate cox regression was used to further evaluate survival-related mRNAs, and only mRNA with *P* < 0.05 was selected as the candidate biomarker. Next, the multivariate cox regression was performed on these candidate mRNAs to identify independent prognostic mRNAs, and a risk value was calculated to construct a predictive model for each mRNAs. Based on the above results, the survivalROC R software package was used to plot the receiver operating characteristic (ROC) curve, and the classification model was evaluated according to the area under the curve (AUC). The *t*-test was used to analyze the relationship between candidate prognosis genes and clinical and biological characteristics.

### 2.7. Data Statistics and Visualization

All data analyses were performed using the R software version 3.5.2. The STRING (https://string-db.org/) and the Cytoscape software were used to construct PPI network. The survival curve was drawn by Kaplan-Meier, and the difference significance was evaluated by log-rank test. *P* < 0.05 is considered as statistically significant.

## 3. Results

### 3.1. Basal Breast Cancer Patients Have Higher mRNAsi Values

**Figure 1:**
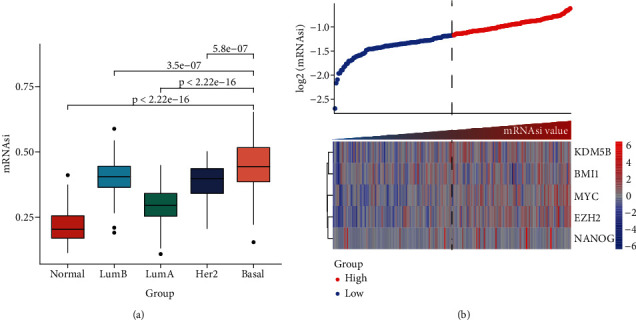
The distribution of tumor stemness index mRNAsi in the basal subtype breast cancer patients. (a) The box plot shows the distribution of tumor stemness index mRNAsi in different breast cancer subtypes and adjacent normal tissues. (b) The expression heat map shows the relationship between tumor stemness index mRNAsi and tumor stem cell markers in patients with the basal breast cancer.

The mRNAsi as the quantization value of stemness index can characterize the similarity degree between tumor cells and stem cells. We here obtained the mRNAsi value of 190 basal, 82 Her2, 566 luminal A, and 214 luminal B patient samples as well as136 normal controls and found that significant differences exist in the mRNAsi value of different breast cancer subtypes. Our results indicated that the mRNAsi values of various breast cancer subtypes are significantly increased than the control group (Figure 1(a)), implying that the cells in tumor tissues are more similar to stem cells than the control group. Obviously, the source of the increase in stemness index is tumor cells. Especially, the basal subtype has the highest mRNAsi, followed by luminal B, Her2, and luminal A (Figure 1(a)). Remarkably, the luminal A patient with the best prognosis has the lowest mRNAsi, but the other three subtype patients with poorer prognosis have higher mRNAsi (Figure 1(a)). Furthermore, we sorted the basal breast cancer patients from low to high according to their mRNAsi values and found that patients with high mRNAsi are usually accompanied by high expression of tumor stem-related genes such as *KDM5B*, *BMI1*, *MYC*, and *EZH2* (Figure 1(b)). These results implied that tumor stem cells might play an important role in the occurrence and development of the basal breast cancer patients.

### 3.2. Identification of mRNAsi-Related Gene Modules

**Figure 2:**
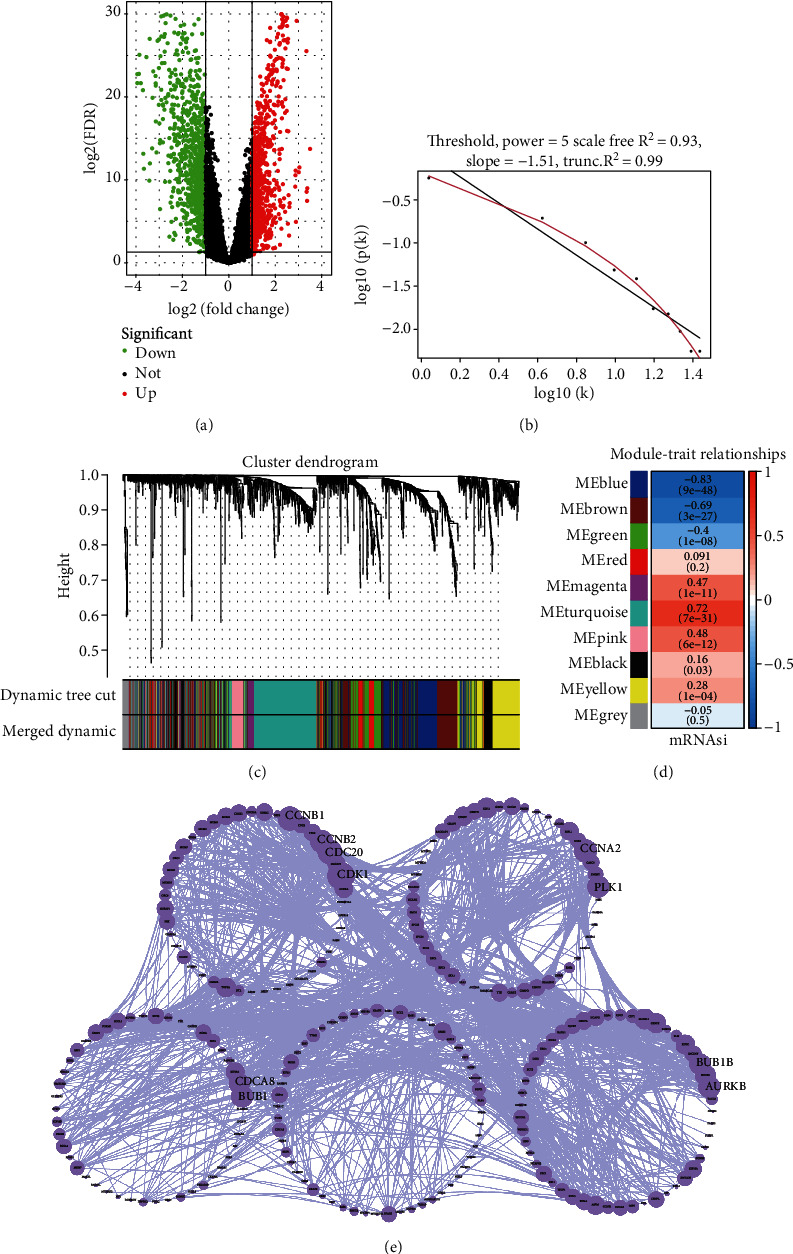
Gene modules are related to tumor stemness index mRNAsi in patients with basal subtype. (a) The volcano map shows the distribution of differentially expressed genes in the adjacent normal tissues of patients with basal subtype. (b) Linear fitting curve under certain soft threshold conditions. The soft threshold *β* = 5 is used to realize the scale-free topology criterion of the network. (c) The clustering dendrogram of differentially expressed genes is based on the tumor stemness index mRNAsi. Each branch represents a gene, each color represents a coexpression module, and gray represents genes that cannot be clustered into modules. (d) The heat map shows the correlation between the different modules and the tumor stemness index mRNAsi. (e) Protein interaction network analysis of mRNAsi-related genes. The size of the circle represents the centrality of the gene that refers to the number of other genes connected to the gene. The larger the number, the larger the circle.

Because the mRNAsi value of the basal subtype patients is the highest among all five breast cancer subtypes, our study thus focused on the basal subtype patients. Herein, we calculated and acquired 1,785 differentially expressed genes containing 880 upregulated and 905 downregulated genes (Figure 2(a)). These genes were further clustered to similar modules by using the WGCNA. We chose *β* value = 5 to establish the proximity matrix so that gene distribution conforms to the scale-free network (Figure 2(b)). After determining *β* value, we continued to test whether the network under the selected *β* value was in line with the scale-free network distribution standard. Remarkably, the number of linked genes varies exponentially in the scale-free topology, so the distribution in the log chart is linear. Our results demonstrated that the closer the fitting value *R*^2^ is to 1, the better the correlation between genes is (Figure 2(b)), indicating that the network is more compliance with the scale-free network distribution. Additionally, we found that the constructed network *K* is significant negative correlation with *P* (*K*) (*R*^2^ = 0.93), which revealed that the selected *β* value can establish the gene scale-free network (Figure 2(b)). We next used the cluster dendrogram to group coexpressing genes into various modules shown in different colors (Figure 2(c)). After cutting and merging by the hybrid dynamic cutting tree algorithm in Figure 2(c), we further extracted the 10 effective modules (Figure 2(d)) and evaluated the significance values of 10 modules through the correlation between each module and mRNAsi (Figure 2(d)). Interestingly, we found that the most significant positive correlation exists between the turquoise module and mRNAsi (*r* = 0.72, *P* < 0.0001), whereas the highest negative correlation exists between the blue module and mRNAsi (*r* = −0.83, *P* < 0.0001) (Figure 2(d)). We thus selected the genes of the turquoise module for in-depth study. In the turquoise module, we found that a total of 385 differentially expressed genes are highly correlated with mRNAsi. We next performed protein network analysis on the 385 genes and found that extensive protein interactions exist in the turquoise module (Figure 2(e)). We further extracted the top 10 hub proteins such as CDK1, CDC20, CCNB2, CCNB1, CDCA8, BUB1, CCNA2, PKL1, BUB1B, and AURK from the PPI network (Figure 2(e)). Interestingly, these proteins are intensively involved in the cell cycle and mitosis process [32–34], implying that the cell cycle might be related to the higher tumor stemness of the basal subtype patients.

### 3.3. GO and KEGG Enrichment Analysis of mRNAsi-Related Modular Genes

**Figure 3:**
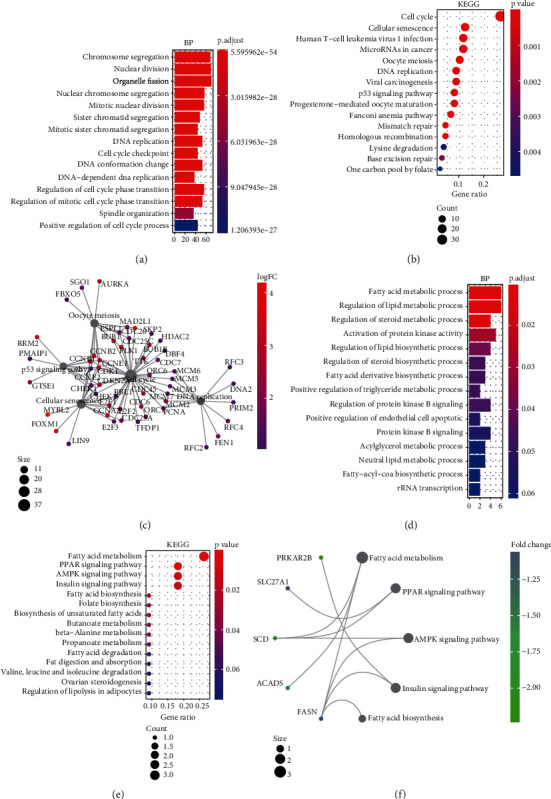
Functional enrichment analysis of tumor stemness index mRNAsi-related genes. (a) Enrichment analysis of biological processes involved in upregulated tumor stemness index-related genes. (b) Enrichment analysis of KEGG signaling pathway involved in upregulated tumor stemness index-related genes. (c) The top 5 KEGG network graphs enriched by the upregulated tumor stemness index-related genes. (d) Enrichment analysis of the biological processes involved in downregulated tumor stemness index-related genes. (e) Enrichment analysis of KEGG signaling pathway involved in downregulated tumor stemness index-related genes. (f) The top 5 KEGG network graphs enriched by downregulated tumor stemness index-related genes.

To understand the roles of these mRNAsi-related genes in the basal subtype, we used GO and KEGG functional analysis to explore how these genes influence the occurrence and development of the basal subtype patients. We here subdivided these genes into upregulated and downregulated genes. GO results demonstrated that these upregulated genes are mainly enriched in biological processes (BP) such as chromosome segregation, nuclear division, organelle fission, sister chromatid separation, DNA replication, and cell cycle checkpoint (Figure 3(a)), while KEGG analysis results indicated that these upregulated genes can participate in the cell cycle, cell aging, DNA replication, p53 signaling pathway, mismatch repair, homologous recombination, and so on (Figure 3(b)). The network diagram displayed the top 5 signal pathways containing upregulated genes (Figure 3(c)). Especially, the upregulated expressions of many genes of the top 5 signal pathways, such as *CCNB1*, *CDC20*, *CDK1*, *CDC25C*, *CHEK2*, *MYBL2*, *E2F1*, *E2F2*, and *FOXM1* (Figure 3(c)), have been reported to be related to breast cancer occurrence [35–38], implying that the upregulated genes might be related to the more vigorous proliferation of tumors and stem cell as well as deterioration. In contrast, these downregulated genes are mainly enriched in fatty acid metabolism, liposome metabolism, protein kinase activity biological processes (Figure 3(d)) and PPAR, AMPK, insulin, and fatty acid metabolism signaling pathways (Figure 3(e)), while the KEGG network diagram showed that these downregulated genes involved in these signaling pathways are mainly P*RKAR2B*, *SLC27A1*, *SCD*, *ACADS*, and *FASN* (Figure 3(f)). This seemed to imply that these downregulated genes could lead to cancer stem cells to weaken high-energy metabolism and turn to low-energy anaerobic metabolism. Taken together, our results suggested that the high tumor stemness in the basal subtype patients might be driven by the abnormal cell cycle, activation of cell division, and metabolic reprogramming.

### 3.4. Identifying Prognostic-Related Genes in the mRNAsi-Related Modules

**Figure 4:**
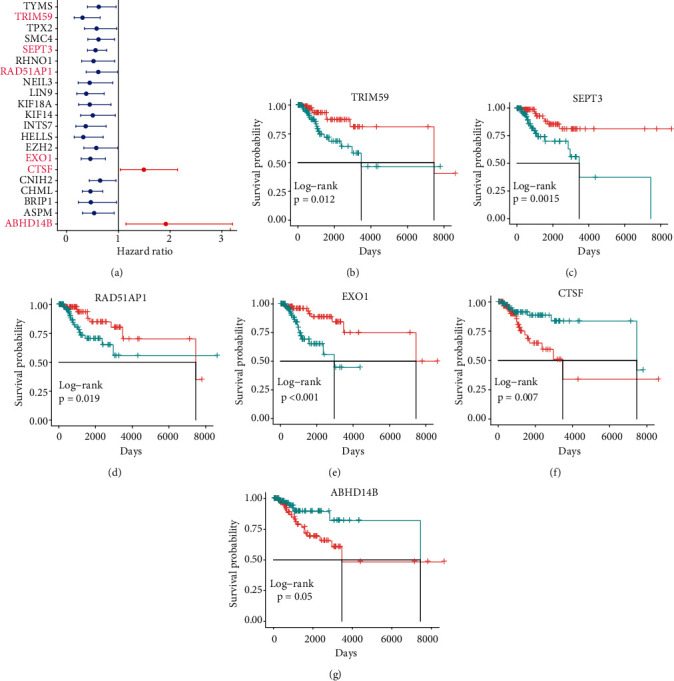
Identification of prognostic-related genes related to tumor stemness index. (a) The forest plot shows the prognostic-related genes screened by single factor cox regression analysis. (b–g) Graphs of overall survival rate of 6 independent prognostic tumor stemness index-related genes. The 6 genes are TRIM59, SEPT3, RAD51AP1, EXO1, CTSF, and ABHD14B.

In order to identify which of the mRNAsi-related genes can be used for prognostic prediction of patients, we further used a two-step analysis to identify candidate genes. We firstly used the univariate cox regression analysis to screen 21 potential candidate genes (Figure 4(a)). Remarkably, only *CTSF* and *ABHD4B* of them are risk factors (HR > 1), whereas the rest 19 genes are all protective factors (HR < 1) (Figure 4(a)). We next used the multivariate cox regression analysis to find that *TRIM59*, *SEPT3*, *RAD51AP1*, and *EXO1* can act as independent prognostic protective factors, as well as *CTSF* and *ABHD4B* can serve as independent prognostic bad factors. Especially, the survival curves of these 6 prognostic genes demonstrated that the basal patients with highly expressed *TRIM59*, *SEPT3*, *RAD51AP1*, and *EXO1* have a higher survival rate than ones with low expression, but the basal patients with highly expressed *CTSF* and *ABHD14B* have a lower survival rate than ones with low expression (Figures 4(b)–4(g)). Our findings revealed that the 6 genes might be related to cancer stem cells and serve as the prognostic markers for the basal breast cancer patients.

### 3.5. Establishing the Prognostic Model for the Basal Patients

**Figure 5:**
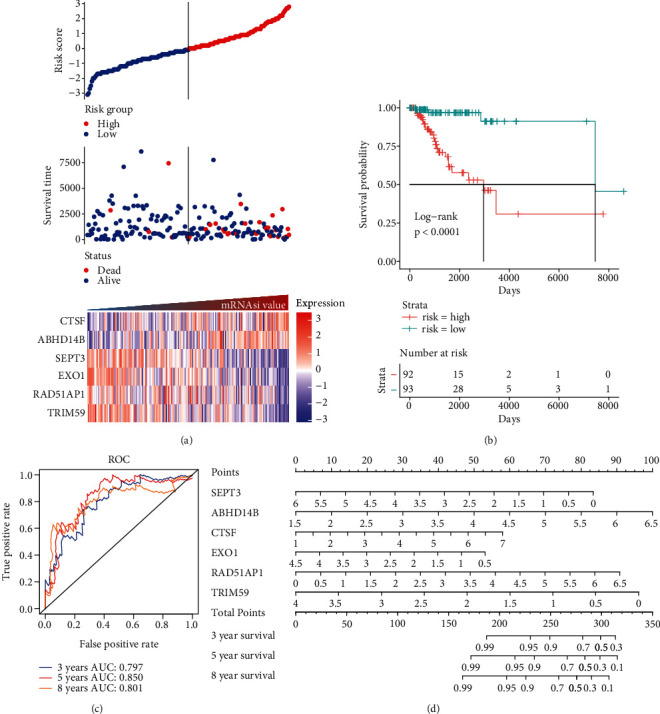
The prognostic value of a prognostic model based on 6 mRNAsi-related genes. (a) The high- and low-risk graph shows the relationship between the expression of prognostic genes and the death density of patients with basal subtypes and the risk value. (b) The overall survival rate curve of basal patients in the high- and low-risk groups. (c) The receiver operating characteristic curve (ROC) shows the accuracy of the model for different years. (d) The nomogram shows how the 6-gene model predicts the overall survival rate of patients at different ages.

Herein, we further detected whether the multigene model composed of the above 6 mRNAsi-related prognostic genes can effectively predict the survival rate of the basal patients. Our results demonstrated that as the increase of the basal patient's risk score, the death density of the basal patients is correspondingly increased (Figure 5(a)), especially the expression levels of genes as protective factors are gradually decreased, but the expressions of genes as risk factors are gradually increased (Figure 5(a)). Furthermore, we divided patients into the high-risk group and the low-risk group and found that the survival rate of patients in the high-risk group is nearly 2 times lower than that in the low-risk group (*P* < 0.0001) (Figure 5(b)). In order to verify the accuracy of the model, we herein drew the ROC curve based on the 6 genes as a signature. Generally, an AUC value greater than 0.6 means that the model has a certain prognostic value, and the larger the value is, the more accurate it is, but an AUC value less than 0.5 means that the model is meaningless. Our results demonstrated average 3-, 5-, and 8-year AUC values for 0.797, 0.850, and 0.801, respectively (Figure 5(c)), indicating that the prognostic model has a good predictive effect on the survival rates of the basal patients. Remarkably, we further constructed the nomogram model based on these 6 genes to predict the survival rate of patients (Figure 5(d)). Interestingly, we can get the score sum corresponding to each gene according to the expression of the 6 genes in the basal patients through this prediction model, which can be used to predict the survival rate of patients in different time periods, and be helpful for the clinical monitoring and management of the basal patients.

### 3.6. Relationship between 6 Prognostic Genes and Clinical Factors

In order to explore how these 6 genes affect the clinical indicators of the basal patients, we further investigated the relationship within the 6 genes and the patient's age, T stage, American Joint Committee on Cancer (AJCC) stage, lymph nodes present (N stage), and tumor bearing status. As shown in Table 1, *SEPT3*, *RAD51AP1*, and *EXO1* are more highly expressed in older patients than young ones, but *TRIM59*, *CTSF*, and *ABHD4B* have no significant changes between them. The expression of *RAD51AP1* is also higher in patients with high T stage than ones with low T stage, but the expression of *ABHD14B* is lower in patients with high T stage, as well as no significant changes in other genes. Similarly, the expressions of *SEPT3*, *RAD51AP1*, and *EXO1* are significantly increased in patients with higher AJCC stage and N stage than ones with lower AJCC stage and N stage, and the rest genes are no significant. While the expression levels of *SEPT3*, *RAD51AP1*, and *EXO1* are higher in tumor-bearing patients, while *ABHD14B* is lower. These results revealed that the 6 prognostic genes are related to the clinical indicators of the basal patients to varying degrees, implying that the 6 genes might play a crucial role in the development and progression of the basal breast cancer.

### 3.7. The Landscape of Immune Infiltrating Cells of High- and Low-Risk Groups

**Figure 6:**
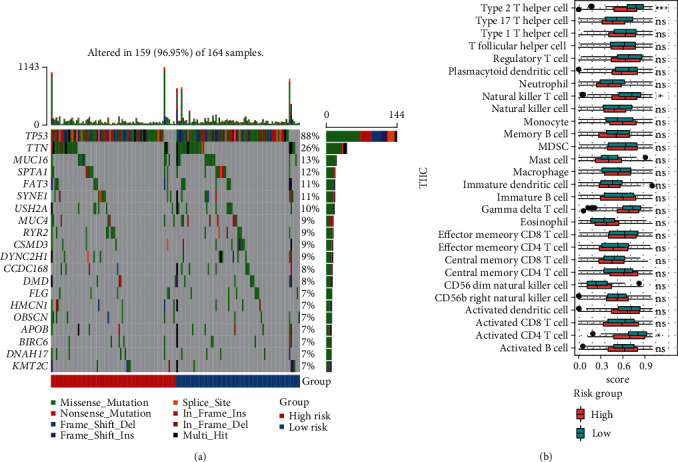
Analysis of mutations and tumor immune infiltrating cells in the basal patients in high- and low-risk groups. (a) The mutation map of the basal patients in the high- and low-risk groups. The top 20 highly mutated genes were displayed. (b) The distribution of tumor infiltrating immune cells in the basal patients in the high- and low-risk groups.

Here, we further explored the underlying factors that cause the difference in the survival rate between the high-risk group and the low-risk group. Because mutations and immune cell infiltrations play very important roles in the treatment and prognosis monitoring of cancer patients, we therefore analyzed mutations and immune cell infiltration of the high-risk group and the low-risk group in the basal patients. Our results demonstrated that the most frequently mutated genes are *TP53*, *TTN*, *MUC16*, *SPTA1*, *FAT3*, *SYNE1*, *USH2A*, and so on in the basal subtype patients, but they have no significant mutation difference between the high-risk and low-risk group (Figure 6(a)). This implied that the difference in survival rate between them is not mainly driven by gene mutation differences. We thereby further calculated the infiltration of 28 kinds of immune cells in tumor tissues. We found that most immune cells have no significant differences, except for type 2 T helper cells, natural killer T cells, and activated CD4 T cells in the high- and low-risk groups; in particular, the infiltration of type 2 T helper cells, natural killer T cells, and activated CD4 T cells in the tumor tissues of patients in the low-risk group is significantly increased than the high-risk group (Figure 6(b)). This implied that these three types of immune infiltrating cells might serve as a protective effect on patients in the low-risk group, thereby increasing the survival rate.

### 3.8. Drug Sensitivity Analysis of Potential mRNAsi-Related Markers

**Figure 7:**
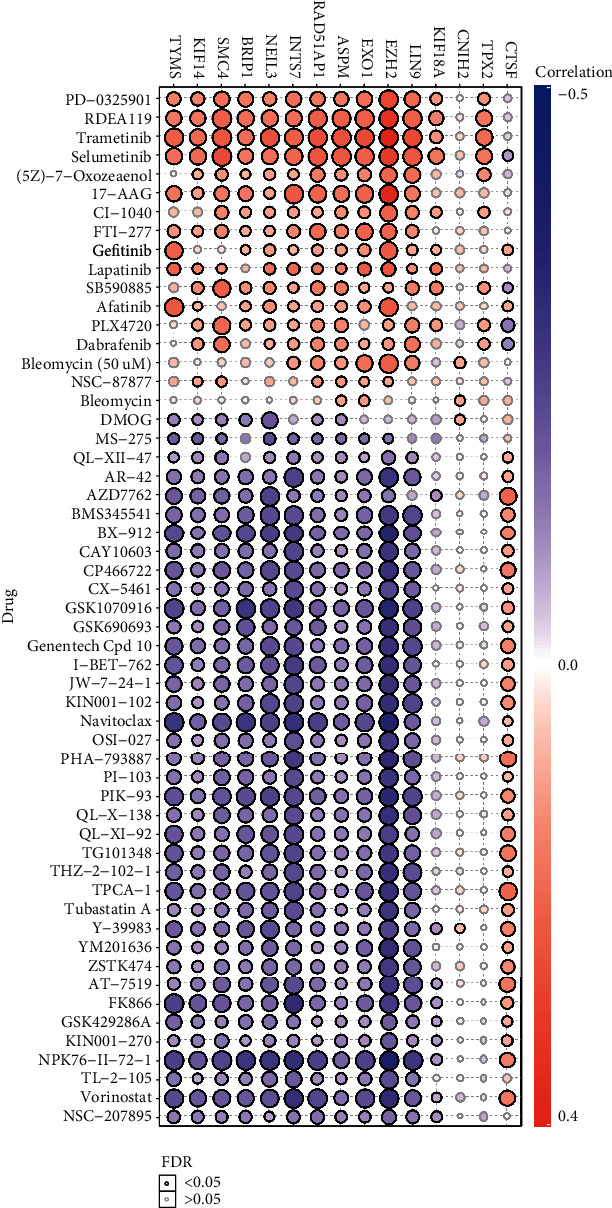
Drug sensitivity analysis of prognostic-related genes. Analysis of drug sensitivity data of 21 genes related to the prognosis of patients with basal subtype. The drug sensitivity data comes from the GSCALite database.

The 21 potential prognostic markers above are closely related to mRNAsi and play very important roles in cancer stem cells of the basal patients. Therefore, finding drug susceptibility data targeting these genes can be helpful for the treatment of the basal breast cancer patients. We herein only obtained drug susceptibility data for 15 potential prognostic genes in the GSCALite database (Figure 7). Interestingly, the three independent prognostic-related genes (*RAD51AP1*, *EXO1*, *CTSF*) have drug susceptibility data; in particular, both *RAD51AP1* and *EXO1* present high drug resistance to PD-0325901, RDEA119, trametinib, selumetinib, and so on (Figure 7). Remarkably, these drugs are all inhibitors that target MEK signaling [39–41], which suggested that these drug might not be suitable for the treatment of the basal breast cancer patients. On the contrary, the most sensitive drugs for *RAD51AP1* and *EXO1* are Navitoclax, NPK76-II-72-1, and Vorinostat (Figure 7). Especially, Navitoclax is an effective Bcl-2 inhibitor, while NPK76-II-72-1 is a kinase inhibitor, as well as Vorinostat is an HDAC inhibitor [42, 43]. We thus suggested that the basal breast cancer patients should be able to obtain good therapeutic effect via using these drugs. Additionally, these insensitive drugs for *CTSF* gene include AZD7762, PHA-793887, TPCA-1, and so on (Figure 7). AZD776 is an ATP-competitive checkpoint kinase inhibitor, and PHA-793887 is a new effective inhibitor of CDK2, CDK5, and CDK7, as well as TPCA-1 can target signals such as IKB/IKK. It is worth noting that *CTSF* seems to be resistant to most drugs, except for PLX4720 and Dabrafenib that are Raf inhibitors. Our findings suggested that these drug sensitivity data for cancer stem-related genes could be helpful for improving the efficacy and prognosis of the basal subtype patients.

## 4. Discussion

Breast cancer is a kind of tumor with high heterogeneity [44]. Especially, the basal breast cancer is the subtype with the worst prognosis due to factors such as rapid progression, high metastasis, high recurrence, and drug resistance [45, 46].

Tumor stem cells are the source of unlimited proliferation and recurrence of malignant tumors. Tumor stem cells play very crucial roles in a variety of malignant phenotypes, such as the proliferation, invasion, and invasion of tumor cells. Tumor stem cells have the characteristics of helping tumor metastasis and drug resistance. Tumor stem cells have become the research hotspot in the field of cancer in recent years [47]. Tumor stem cells provide a new understanding on elucidating the occurrence and development of cancer and a new idea for targeted therapy for cancer [18]. Currently, although cancer stem cells are yet not reported to be an important component of the basal breast cancer, they have been demonstrated to be related to the occurrence, development, drug resistance, recurrence, and metastasis [18, 48, 49]. However, due to the lack of a unified description on cancer stem cells, the clinical application of cancer stem cell therapy has still to be developed, and the personalized treatment of the basal breast cancer has made slow progress. Interestingly, mRNAsi as an index to quantify the tumor stemness of tumor patients has recently been developed through computational biology and bioinformatics [19, 20], which makes it convenient to explore genes related to tumor stemness.

In this study, we found that patients with the basal subtype breast cancer have higher tumor stemness (mRNAsi score) (Figure 1(a)), implying that mRNAsi might be related to the poor prognosis of patients with the basal subtype. We identified 358 genes with highly positive correlation with mRNAsi (Figure 2(d)), and many genes of them exist in strong interaction from the PPI network (Figure 2(e)), as well as these hub gproteins CDK1, CDC20, CCNB1, CDK1, CDC20, and CCNB1 are, respectively, significantly associated with cell cycle (Figure 2(e)). Remarkably, previous studies have revealed that the upregulated expressions of *CDK1* and *CCNB1* are correlated with the worse OS in the basal subtype breast cancer, while *CDC20*, *CCNB1*, and *CDK1* could act as diagnostic and prognostic markers in breast cancer. Especially, *FOXM1* and *E2F1* have also been demonstrated to be significantly associated with CSCs in a variety of tumors [50–52]. Taken together, we suggested that the mRNAsi-related genes could play very important roles in the occurrence and development as well as prognosis of the basal breast cancer patients.

Herein, we found that the upregulated mRNAsi-related genes are significantly enriched in the cell cycle, cell division, DNA replication, and p53 signaling pathway (Figure 3). Many studies have revealed that abnormality of these pathways can be the basis for tumor stem cells to maintain rapid proliferation and invasion [53–56]. Interestingly, some genes involved in these processes and signal pathways, such as *CCNB1*, *CDK1*, *CHEK2*, and *MYBL2*, have been widely reported as oncogenes and used in the clinical diagnosis for breast cancer patients [35, 38]. Especially, studies have demonstrated that some transcription factors (e.g., E2F1, E2F2, and FOXM1) could serve as oncogenes to drive the development and progression of multiple cancers [36, 37]. In our work, we also found that some mRNAsi-related oncogenic transcription factors, such as E2F1, E2F2, and FOXM1, might play an important role in cell cycle activation of the basal subtype (Figure 3(c)), implying that repressing the expression of these oncogenic transcription factors may be beneficial for treating patients with the basal subtype. In contrast, the downregulated mRNAsi-related genes are mainly enriched in metabolic processes and metabolic pathways, such as *PPKAR2B*, *SLC27A1*, *SCD*, *FASN*, and *ACADS*. Tumor cells, especially tumor stem cells, need to provide themselves with a large amount of ATP and other energy substances through metabolic reprogramming, which is one of the characteristics of tumor deterioration [57, 58]. This indicated that the downregulated mRNAsi-related genes can play a key role in the metabolism of cancer stem cells in patients with the basal subtype.

In this study, we identified 21 potential markers for the survival and prognosis of patients with the basal subtype (Figure 4). Especially, we found that *TRIM59*, *SEPT3*, *RAD51AP1*, and *EXO1* can serve as independent protective factors and *CTSF* and *ABHD14B* as the risk factors (Figure 4), and they are also closely related to the clinical indicators of patients with the basal subtype (Table 1). Surprisingly, the prediction model containing the 6 mRNAsi-related genes as a signature could act as an effectively prognostic factor to promote the survival (Figures 5(b) and 5(c)) and could effectively predict the 3-year, 5-year, and 8-year survival rates of patients with the basal subtype (Figures 5(b)–5(d)). More importantly, our findings reveal the survival differences of patients between the high-risk group and low-risk group are mainly driven by differences in immune infiltrating cells (e.g., type 2 helper T cells, activated CD4 T cells, and NKT cells), rather than differences in gene mutations (Figure 6). Remarkably, we here found that 15 mRNAsi-related genes could be helpful for the treatment of the basal subtype patients. Our results revealed that the 15 genes are resistant to multiple drugs such as Navitoclax, NPK76-II-72-1, and Vorinostat (Figure 7). These genetically sensitive drugs include BX-912, Navitociax, and NPK76-II-72-1. Especially, this *CTSF* gene as a risk factor is resistant to a variety of tested drugs, except for selumetinib, SB590885, PLX4720, and Dabrafenib (Figure 7). We thereby suggested that the widespread resistance of this *CTSF* gene may be a potential reason impacting the treatment effect of the basal breast cancer patients, and selumetinib, SB590885, PLX4720, and Dabrafenib can be considered as potential therapy or adjuvant therapy drugs because it has a good effect on the basal breast cancer patients.

In summary, our study has revealed that the basal subtype breast cancer has a higher mRNAsi score, which indicates that this subtype can be driven by cancer stem cells. Our results demonstrate mRNAsi-related modular genes mainly involved in cell cycle activation and metabolic reprogramming, which may be internal factors that maintain the survival of the basal breast tumor malignant stem cells. We construct a prediction model containing the 6 mRNAsi-related prognostic genes, which can be used to predict the survival rate of the basal breast cancer patients. We provide the drug sensitivity data of 15 genes related to mRNAsi, which will be helpful for the treatment of the basal breast cancer patients.

## Figures and Tables

**(a):** 

Parameters		*N*	TRIM59		SEPT3		RAD51AP1	
			M ± SD	*P* value	M ± SD	*P* value	M ± SD	*P* value
Age (*n* = 185)	≤50	73	1.70 ± 0.55	0.151	2.79 ± 1.34	0.044	3.28 ± 0.96	0.022
	>50	112	1.86 ± 0.51		3.30 ± 1.16		3.58 ± 0.56	

T (*n* = 184)	T3-T4	23	1.75 ± 0.55	0.103	2.94 ± 1.25	0.152	3.40 ± 0.90	0.010
	T1-T2	161	1.57 ± 0.53		2.45 ± 1.69		2.89 ± 0.92	

Stage (*n* = 182)	III-IV	29	1.74 ± 0.53	0.333	2.98 ± 1.30	0.010	3.38 ± 0.91	0.012
	I-II	153	1.61 ± 0.59		2.21 ± 1.35		2.89 ± 0.86	

N (*n* = 185)	N2-N3	21	1.74 ± 0.55	0.459	3.00 ± 1.28	0.002	3.38 ± 0.93	0.040
	N0-N1	164	1.65 ± 0.55		2.10 ± 1.33		3.02 ± 0.79	

Status (*n* = 179)	With tumor	25	1.74 ± 0.52	0.093	2.96 ± 1.32	0.019	3.36 ± 0.86	<0.001
	Tumor free	154	1.48 ± 0.69		2.16 ± 1.45		2.92 ± 1.17	

**(b):** 

Parameters		*N*	EXO1		CTSF		ABHD14B	
			M ± SD	*P* value	M ± SD	*P* value	M ± SD	*P* value
Age (*n* = 185)	≤50	73	2.89 ± 0.76	0.001	4.21 ± 1.03	0.138	3.95 ± 0.72	0.300
	>50	112	3.37 ± 0.65		3.96 ± 0.76		4.07 ± 0.54	

T (*n* = 184)	T3-T4	23	3.00 ± 0.73	0.139	4.14 ± 0.98	0.225	3.92 ± 0.71	0.049
	T1-T2	161	2.73 ± 0.90		4.40 ± 1.05		4.16 ± 0.55	

Stage (*n* = 182)	III-IV	29	3.02 ± 0.72	0.025	4.17 ± 1.02	0.709	3.94 ± 0.72	0.218
	I-II	153	2.59 ± 0.87		4.24 ± 0.89		4.10 ± 0.59	

N (*n* = 185)	N2-N3	21	3.02 ± 0.73	0.031	4.12 ± 0.99	0.128	3.94 ± 0.72	0.170
	N0-N1	164	2.64 ± 0.83		4.44 ± 0.98		4.10 ± 0.53	

Status (*n* = 179)	With tumor	25	3.03 ± 0.76	0.001	4.13 ± 1.00	0.261	3.90 ± 0.69	0.045
	Tumor free	154	2.43 ± 0.66		4.38 ± 0.96		4.24 ± 0.74	

The abbreviations are as follows, which are derived from the identification of the patients according to the guidelines of the American Joint Committee on Cancer (AJCC). Age: the age of the patient at the time of first diagnosis; T: extent of the primary cancer when the patient was first diagnosed; stage: the extent of a cancer that whether the disease has spread from the original site to other parts of the body; N: extent of the regional lymph nodes present for the cancer at the time of initial diagnosis; status: the neoplasm cancer status when the patient was first diagnosed. For specific clinical staging information of basal breast cancer samples, please refer to Supplementary Table S1.

## Data Availability

The data links and sources used to support the results of this study have been included in the article.

## Supplementary Materials

Supplementary Materials Table S1: specific clinical staging information of basal breast cancer samples.
