# Supplementary material for: Analyzing mRNAsi-Related Genes Identifies Novel Prognostic Markers and Potential Drug Combination for Patients with Basal Breast Cancer
Source: Dis Markers. 2021 Oct 4;2021:4731349. doi: 10.1155/2021/4731349 (PMC8505092; doi:10.1155/2021/4731349)
Supplement: Supplementary Materials — Table S1: specific clinical staging information of basal breast cancer samples. [file 4731349.f1.zip › Table S1.pdf]

Table S1: Specific clinical staging information of basal breast cancer samples.

SampleID	Age	pathologic_N	pathologic_T	pathologic_stage	Neoplasm_cancer_status	Outcome	OS
TCGA-A1-A0SK-01	54	N0 (i-)	T2	Stage IIA	WITH TUMOR	1	967
TCGA-A1-A0SO-01	67	N1	T2	Stage IIB	TUMOR FREE	0	852
TCGA-A1-A0SP-01	40	N0 (i-)	T2	Stage IIA	TUMOR FREE	0	584
TCGA-A2-A04P-01	36	N3c	T2	Stage IIIC	WITH TUMOR	1	548
TCGA-A2-A04Q-01	48	N0 (i-)	T1	Stage IA	TUMOR FREE	0	2385
TCGA-A2-A04T-01	62	N0 (i-)	T2	Stage IIA	TUMOR FREE	0	2246
TCGA-A2-A04U-01	47	N0 (i+)	T2	Stage IIA	TUMOR FREE	0	2654
TCGA-A2-A0CM-01	40	N0 (i-)	T2	Stage IIA	WITH TUMOR	1	754
TCGA-A2-A0D0-01	60	N0 (i-)	T2	Stage IIA	TUMOR FREE	0	2048
TCGA-A2-A0D2-01	45	N0 (i+)	T2	Stage IIA	TUMOR FREE	0	1027
TCGA-A2-A0ST-01	62	N1a	T1c	Stage IIA	WITH TUMOR	0	3017
TCGA-A2-A0SX-01	48	N0 (i-)	T1c	Stage IA	WITH TUMOR	0	1534
TCGA-A2-A0T0-01	59	N1	T2	Stage IIB	TUMOR FREE	0	533
TCGA-A2-A0T2-01	66	N3	T3	Stage IV	WITH TUMOR	1	255
TCGA-A2-A0YE-01	48	N1a	T2	Stage IIB	TUMOR FREE	0	554
TCGA-A2-A0YJ-01	39	N2a	T3	Stage IIIA	WITH TUMOR	0	566
TCGA-A2-A0YM-01	67	N0 (i-)	T2	Stage IIA	TUMOR FREE	0	965
TCGA-A2-A1G1-01	85	N1	T2	Stage IIB	TUMOR FREE	0	584
TCGA-A2-A25F-01	66	N0 (i-)	T2	Stage IIA	TUMOR FREE	0	322
TCGA-A2-A3XS-01	62	N2a	T1	Stage IIIA	WITH TUMOR	1	1032
TCGA-A2-A3XT-01	45	N1a	T2	Stage IIB	TUMOR FREE	0	2770
TCGA-A2-A3XU-01	35	N1mi	T2	Stage IIB	WITH TUMOR	1	912
TCGA-A2-A3XX-01	49	N0	T2	Stage IIA	TUMOR FREE	1	1439
TCGA-A2-A3XY-01	49	N1a	T2	Stage IIB	WITH TUMOR	1	1093
TCGA-A2-A3Y0-01	57	N1a	T2	Stage IIB	TUMOR FREE	0	1546
TCGA-A2-A4RX-01	67	N0	T2	Stage IIA	TUMOR FREE	0	742
TCGA-A2-A4S1-01	66	N0	T2	Stage IIA	TUMOR FREE	0	820
TCGA-A7-A0CE-01	57	N0 (i-)	T2	Stage IIA	TUMOR FREE	0	1074
TCGA-A7-A0DA-01	62	N0 (i-)	T2	Stage IIA	TUMOR FREE	0	1085
TCGA-A7-A13D-01	46	N0 (i-)	T2	Stage IIA	TUMOR FREE	0	965
TCGA-A7-A13E-01	62	N1	T2	Stage IIB	WITH TUMOR	1	614
TCGA-A7-A26G-01	50	N0 (i+)	T2	Stage IIA	TUMOR FREE	0	722
TCGA-A7-A4SD-01	52	N0	T2	Stage IIA	TUMOR FREE	0	441
TCGA-A7-A4SE-01	54	N0	T2	Stage IIA	TUMOR FREE	0	644
TCGA-A7-A5ZV-01	62	N0	T2	Stage IIA	TUMOR FREE	0	368
TCGA-A7-A6VV-01	51	N0	T2	Stage IIA	TUMOR FREE	0	313
TCGA-A7-A6VW-01	48	N0	T2	Stage IIA	TUMOR FREE	0	285
TCGA-A7-A6VY-01	48	N1	T2	Stage IIB	TUMOR FREE	0	266
TCGA-A8-A07C-01	57	N0	T2	Stage IIA	TUMOR FREE	0	1034
TCGA-A8-A07O-01	51	N0	T2	Stage IIA	TUMOR FREE	0	304
TCGA-A8-A07R-01	80	N3a	T2	Stage IIIC	NA	0	273
TCGA-A8-A07U-01	66	N2a	T2	Stage IIIA	TUMOR FREE	0	760
TCGA-A8-A08R-01	52	N1a	T2	Stage IIB	TUMOR FREE	0	30
TCGA-AC-A2BK-01	78	N2a	T2	Stage IIIA	TUMOR FREE	0	2222
TCGA-AC-A2QJ-01	48	N0	T4b	Stage IIIB	WITH TUMOR	1	446
TCGA-AC-A62X-01	72	N0	T2	Stage IIA	TUMOR FREE	0	417
TCGA-AC-A6IW-01	73	N0 (i-)	T2	Stage IIA	TUMOR FREE	0	413
TCGA-AC-A7VC-01	56	N0	T3	Stage IIB	TUMOR FREE	0	1
TCGA-AC-A8OQ-01	72	N1a	T2	Stage IIB	TUMOR FREE	0	34
TCGA-AN-A04D-01	58	N1	T2	Stage IIB	TUMOR FREE	0	52
TCGA-AN-A0AL-01	41	N0	T4	Stage IIIB	TUMOR FREE	0	227
TCGA-AN-A0AR-01	55	N0	T2	Stage IIA	TUMOR FREE	0	10
TCGA-AN-A0AT-01	62	N0	T2	Stage IIA	TUMOR FREE	0	10
TCGA-AN-A0FJ-01	59	N2	T2	Stage IV	TUMOR FREE	0	242
TCGA-AN-A0FL-01	62	N0	T2	Stage IIA	TUMOR FREE	0	231
TCGA-AN-A0FX-01	52	N0	T2	Stage IIA	TUMOR FREE	0	10

TCGA-AN-A0G0-01	56	N0	T2	Stage IIA	TUMOR FREE	0	16
TCGA-AN-A0XU-01	54	N0	T2	Stage IIA	TUMOR FREE	0	10
TCGA-AO-A0J4-01	41	N0 (i-)	T1c	Stage IA	TUMOR FREE	0	1587
TCGA-AO-A0J6-01	61	N0 (i-)	T2	Stage IIA	TUMOR FREE	0	1140
TCGA-AO-A0JL-01	59	N2a	T2	Stage IIIA	TUMOR FREE	0	1683
TCGA-AO-A124-01	38	N0 (i-)	T2	Stage IIA	TUMOR FREE	0	3506
TCGA-AO-A128-01	61	N0 (i-)	T2	Stage IIA	TUMOR FREE	0	3248
TCGA-AO-A129-01	29	N1a	T2	Stage IIB	TUMOR FREE	0	3286
TCGA-AO-A12F-01	36	N0 (i-)	T2	Stage IIA	TUMOR FREE	0	1842
TCGA-AO-A1KR-01	51	N0	T2	Stage IIA	TUMOR FREE	0	2513
TCGA-AQ-A04J-01	45	N0 (i+)	T2	Stage IIA	TUMOR FREE	0	819
TCGA-AQ-A54N-01	51	N0	T2	Stage IIA	TUMOR FREE	0	78
TCGA-AR-A0TP-01	43	N0	T2	Stage IIA	TUMOR FREE	0	4275
TCGA-AR-A0TS-01	46	N1	T2	Stage IIB	TUMOR FREE	0	2558
TCGA-AR-A0TU-01	35	N0	T2	Stage IIA	TUMOR FREE	0	709
TCGA-AR-A0U0-01	73	N1	T2	Stage IIB	TUMOR FREE	0	1988
TCGA-AR-A0U4-01	54	N0	T2	Stage IIA	TUMOR FREE	0	3261
TCGA-AR-A1AH-01	51	N1	T2	Stage IIB	TUMOR FREE	0	3807
TCGA-AR-A1AI-01	47	N0	T2	Stage IIA	TUMOR FREE	0	3296
TCGA-AR-A1AJ-01	83	N0	T1	Stage I	TUMOR FREE	0	3072
TCGA-AR-A1AQ-01	49	N0	T2	Stage IIA	TUMOR FREE	0	3021
TCGA-AR-A1AR-01	50	N2	T1	Stage IIIA	WITH TUMOR	1	524
TCGA-AR-A1AY-01	65	N0	T1	Stage I	TUMOR FREE	0	1026
TCGA-AR-A24Q-01	49	N0	T3	Stage IIB	TUMOR FREE	0	3172
TCGA-AR-A251-01	51	N2	T2	Stage IIIA	TUMOR FREE	0	3030
TCGA-AR-A256-01	45	N0	T2	Stage IIA	NA	1	2854
TCGA-AR-A5QQ-01	68	N1	T3	Stage IIIA	WITH TUMOR	1	322
TCGA-B6-A0I1-01	73	N0	T2	Stage IIA	TUMOR FREE	1	2361
TCGA-B6-A0I2-01	45	N0 (i-)	T1c	Stage IA	TUMOR FREE	0	4361
TCGA-B6-A0I6-01	49	N1	T1c	Stage IIA	WITH TUMOR	1	991
TCGA-B6-A0IJ-01	42	N0 (i-)	T3	Stage IIB	TUMOR FREE	0	7106
TCGA-B6-A0IQ-01	40	N1b	T3	Stage IIIA	TUMOR FREE	0	4285
TCGA-B6-A0RE-01	61	N0 (i-)	TX	Stage X	TUMOR FREE	0	7777
TCGA-B6-A0RT-01	39	N1	T3	Stage IIIA	TUMOR FREE	0	2721
TCGA-B6-A0RU-01	40	N0 (i-)	T1c	Stage IA	TUMOR FREE	0	8605
TCGA-B6-A0WX-01	40	N1b	T3	Stage IIIA	WITH TUMOR	1	639
TCGA-B6-A0X1-01	48	N1	T2	Stage IIB	TUMOR FREE	1	7455
TCGA-B6-A1KF-01	68	N1	T2	Stage IIB	TUMOR FREE	0	3088
TCGA-B6-A3ZX-01	50	N1	T3	Stage IV	WITH TUMOR	1	1152
TCGA-B6-A400-01	43	N2a	T2	Stage IIIA	TUMOR FREE	0	215
TCGA-B6-A402-01	47	N0 (i-)	T1c	Stage I	WITH TUMOR	0	2281
TCGA-B6-A409-01	44	N2a	T1c	Stage IIIA	WITH TUMOR	1	573
TCGA-BH-A0AV-01	52	N0	T1c	Stage I	TUMOR FREE	0	1820
TCGA-BH-A0B3-01	53	N1a	T2	Stage IIB	TUMOR FREE	0	1203
TCGA-BH-A0B9-01	44	N0 (i-)	T1c	Stage IA	TUMOR FREE	0	1572
TCGA-BH-A0BG-01	73	N0	T1	Stage I	TUMOR FREE	0	1871
TCGA-BH-A0BL-01	35	N0	T1c	Stage I	TUMOR FREE	0	2278
TCGA-BH-A0BW-01	71	N0	T1c	Stage I	TUMOR FREE	0	2371
TCGA-BH-A0DL-01	64	N0	T2	Stage IIA	TUMOR FREE	0	2381
TCGA-BH-A0E0-01	38	N3a	T3	Stage IIIC	TUMOR FREE	0	134
TCGA-BH-A0E6-01	69	N0 (i-)	T1c	Stage IA	TUMOR FREE	0	293
TCGA-BH-A0RX-01	59	N0 (i-)	T2	Stage IIA	TUMOR FREE	0	170
TCGA-BH-A0WA-01	82	N0 (i-)	T1c	Stage I	TUMOR FREE	0	701
TCGA-BH-A18G-01	81	N0	T1c	Stage IA	TUMOR FREE	0	149
TCGA-BH-A18Q-01	56	N1b	T2	Stage IIB	NA	1	1692
TCGA-BH-A18T-01	70	N0	T2	Stage IIA	WITH TUMOR	1	224
TCGA-BH-A18V-01	48	N1b	T2	Stage IIB	NA	1	1556
TCGA-BH-A18V-06	48	N1b	T2	Stage IIB	NA	1	1556

TCGA-BH-A1F0-01	80	N1c	T1a	Stage IIA	WITH TUMOR	1	785
TCGA-BH-A1F6-01	51	N2a	T4d	NA	NA	1	2965
TCGA-BH-A1FC-01	78	N1b	T1c	Stage IIA	TUMOR FREE	1	3472
TCGA-BH-A5IZ-01	51	N1a	T2	Stage IIB	TUMOR FREE	0	567
TCGA-C8-A12V-01	55	N0	T2	Stage IIA	TUMOR FREE	0	385
TCGA-C8-A131-01	82	N2	T2	Stage IIIA	TUMOR FREE	0	411
TCGA-C8-A134-01	52	N0	T2	Stage IIA	TUMOR FREE	0	383
TCGA-C8-A1HJ-01	53	N0	T2	Stage IIA	TUMOR FREE	0	5
TCGA-C8-A27B-01	48	N0	T3	Stage IIB	TUMOR FREE	0	439
TCGA-D8-A142-01	74	N0	T3	Stage IIB	TUMOR FREE	0	425
TCGA-D8-A143-01	51	N0	T2	Stage IIA	TUMOR FREE	0	431
TCGA-D8-A147-01	45	N0	T2	NA	TUMOR FREE	0	584
TCGA-D8-A1JK-01	90	N0	T2	Stage IIA	TUMOR FREE	0	612
TCGA-D8-A1JL-01	72	N0	T2	Stage IIA	TUMOR FREE	0	611
TCGA-D8-A1JM-01	59	N1a	T2	Stage IIB	TUMOR FREE	0	590
TCGA-D8-A1XK-01	55	N1a	T2	Stage IIB	TUMOR FREE	0	441
TCGA-D8-A1XQ-01	69	N0	T2	Stage IIA	TUMOR FREE	0	499
TCGA-D8-A27F-01	40	N0	T2	Stage IIA	TUMOR FREE	0	488
TCGA-D8-A27H-01	72	N0	T2	Stage IIA	TUMOR FREE	0	397
TCGA-D8-A27M-01	59	N0	T1c	Stage IA	TUMOR FREE	0	410
TCGA-E2-A14N-01	37	N1	T2	Stage IIB	TUMOR FREE	0	1434
TCGA-E2-A14R-01	62	N0	T2	Stage IIA	TUMOR FREE	0	1174
TCGA-E2-A14X-01	55	N2	T2	Stage IIIA	TUMOR FREE	0	972
TCGA-E2-A14Y-01	35	N0	T2	Stage IIA	TUMOR FREE	0	2109
TCGA-E2-A150-01	48	N0	T2	Stage IIA	TUMOR FREE	0	1935
TCGA-E2-A158-01	43	N1mi	T1c	Stage IIA	TUMOR FREE	0	450
TCGA-E2-A159-01	50	N0	T2	Stage IIA	TUMOR FREE	0	762
TCGA-E2-A1AZ-01	63	N1a	T2	Stage IIB	TUMOR FREE	0	2329
TCGA-E2-A1B6-01	44	N0	T2	Stage IIA	TUMOR FREE	0	867
TCGA-E2-A1II-01	51	N0	T1c	Stage I	TUMOR FREE	0	1025
TCGA-E2-A1LG-01	50	N0	T2	Stage IIA	TUMOR FREE	0	1523
TCGA-E2-A1LH-01	59	N0	T1c	Stage I	TUMOR FREE	0	3247
TCGA-E2-A1LI-01	57	N1	T2	Stage IIB	TUMOR FREE	0	3121
TCGA-E2-A1LK-01	84	N3a	T4b	Stage IIIC	WITH TUMOR	1	266
TCGA-E2-A1LL-01	73	N2a	T3	Stage IIIA	WITH TUMOR	0	1309
TCGA-E2-A1LS-01	46	N0	T1c	Stage IA	TUMOR FREE	0	1604
TCGA-E2-A573-01	48	N0	T1c	Stage IA	TUMOR FREE	0	1062
TCGA-E2-A574-01	44	N0	T2	Stage IIA	TUMOR FREE	0	1179
TCGA-E9-A1N8-01	48	N0	T2	Stage IIA	TUMOR FREE	0	1039
TCGA-E9-A1N9-01	58	N0	T2	Stage IIA	TUMOR FREE	0	1101
TCGA-E9-A1NC-01	61	N1	T2	Stage IIB	TUMOR FREE	0	1203
TCGA-E9-A1ND-01	75	N1	T2	Stage IIB	TUMOR FREE	0	1266
TCGA-E9-A22G-01	47	N0	T2	Stage IIA	TUMOR FREE	0	1239
TCGA-E9-A243-01	52	N0 (i-)	T2	Stage IIA	WITH TUMOR	0	612
TCGA-E9-A244-01	54	N0 (i-)	T2	Stage IIA	TUMOR FREE	0	21
TCGA-E9-A3QA-01	33	N0 (i-)	T2	Stage IIA	TUMOR FREE	0	918
TCGA-E9-A5FL-01	65	N0	T3	Stage IIB	TUMOR FREE	0	24
TCGA-EW-A1OW-01	58	N0	T2	Stage IIA	TUMOR FREE	0	694
TCGA-EW-A1P4-01	43	N0 (i-)	T2	Stage IIA	TUMOR FREE	0	907
TCGA-EW-A1P8-01	58	N3b	T2	Stage IIIC	WITH TUMOR	1	239
TCGA-EW-A1PB-01	70	N1a	T3	Stage IIIA	TUMOR FREE	0	608
TCGA-EW-A1PH-01	52	N1a	T1c	Stage IIA	TUMOR FREE	0	607
TCGA-EW-A3U0-01	61	N1a	T3	Stage IIIA	TUMOR FREE	0	532
TCGA-EW-A6SB-01	62	N0	T2	Stage II	TUMOR FREE	0	760
TCGA-GI-A2C9-01	58	N0	T3	Stage IIB	TUMOR FREE	0	3342
TCGA-GM-A2DF-01	53	N1a	T1c	Stage IIA	TUMOR FREE	0	2155
TCGA-GM-A3XL-01	49	N0 (i-)	T2	Stage IIA	TUMOR FREE	0	2108
TCGA-HN-A2NL-01	56	N0	T2	Stage IIA	TUMOR FREE	0	79

TCGA-LL-A5YP-01	49	N1a	T2	Stage IIB	NA	0	450
TCGA-LL-A6FR-01	50	N0 (i-)	T2	Stage IIA	TUMOR FREE	0	489
TCGA-LL-A73Y-01	67	N0	T1c	Stage IA	TUMOR FREE	0	477
TCGA-LL-A8F5-01	61	N0 (i-)	T2	Stage IIA	TUMOR FREE	0	596
TCGA-OL-A5D7-01	70	N0	T2	Stage IIA	TUMOR FREE	0	1780
TCGA-OL-A5RW-01	40	N1	T1c	Stage IIA	TUMOR FREE	0	1106
TCGA-OL-A5S0-01	66	N1a	T2	Stage IIB	TUMOR FREE	0	620
TCGA-OL-A66I-01	36	N1mi	T1c	Stage IIA	TUMOR FREE	0	714
TCGA-OL-A6VO-01	43	N0	T1c	Stage IA	TUMOR FREE	0	858
TCGA-PL-A8LZ-01	29	N2	T4b	Stage IIIB	TUMOR FREE	0	302
TCGA-S3-AA0Z-01	63	N1a	T2	Stage IIB	TUMOR FREE	0	629
TCGA-S3-AA10-01	65	N0	T2	Stage IIA	TUMOR FREE	0	586
TCGA-S3-AA15-01	51	N1a	T2	Stage IIB	TUMOR FREE	0	525
